# The Management of Pulpless Teeth from the Standpoint of Daily Practice

**Published:** 1888-08

**Authors:** A. W. Harlan

**Affiliations:** Chicago, Ill.


ARTICLE II.

### THE MANAGEMENT OF PULPLESS TEETH FROM THE STANDPOINT OF DAILY PRACTICE.

BY A. W. HARLAN, M. D., D. D. S., CHICAGO, ILL.

It is with no small degree of trepidation that I venture to present to you a few observations on the management of pulpless teeth, in face of the fact of recent contributions to our Society's proceedings and the current periodical literature of the time. However, I hope that the subject itself has enough of interest in it to receive and merit your most thoughtful consideration. In the effort to conserve teeth in the mouth for mastication and articulate speech, the retention of roots for the support of pivot teeth and crowns, and the growing tendency to resort to bridges of the fixed and removable kind, dental surgeons everywhere are devoting much of their thought, ingenuity, and energy in the direction of retaining within the mouth many pulpless teeth, we had presented to us various operative procedures designated by their authors as mechanical methods; others have been urged for adoption as the immediate dressing and filling method, and still others comprising what is known as the lime-dressing or therapeutic expectant method. From the variety of systems proposed in the management of pulpless teeth the student or inexperienced surgeon would hardly be able to choose a safe path in which to tread unless they were directed by a strong hand. Pulpless teeth are found in the mouths of the old, the middle aged, and the young; even the teeth of the first dentition do not escape the ravages of caries, and slow death of the pulp, or its destruction by other means, is not infrequent. Pulpless deciduous teeth and roots may very properly be retained for mastication up to or about the time of the expected eruption of their successors. At this point, however, I will omit their further consideration.

For convenience of treating the subject of the management of pulpless teeth, we will classify them under several headings as follows:

First. Teeth deprived of pulps by means of corrosive medicaments, or immediate operative procedures—cautery, broaches, wooden points, anæsthesia, or accident.

Second. Death of the pulp as a result of capping or filling, from rapid thermal changes.

Third. Death from partial luxation, as from a blow or fall, or, from torsion in correcting irregularities, or from imperfect or partial extractions and replacement of teeth.

Fourth. Death from gradual exposure by caries, or some form of abrasion or erosion.

Fifth. Death from exposure of the pulp through salivary deposits, exposing the apices of roots by displacement of tissues, or from the development of pockets resulting from the so called Riggs' disease and their extension to or around the apices of roots.

Sixth. Death from irritation by pulp-nodules or strangulation, and from unknown causes.

From this classification, imperfect as it is, you will readily agree with me that no single method of operation, surgical or therapeutic treatment, will suffice to meet all indications in the treatment of irritations, inflammation, or abscess. From a somewhat extended experience in the conservation of pulpless teeth of all classes, covering a period of nearly twenty years, in one city, with few birds of passage as clients, my records now show, not hundreds, but thousands of teeth treated and the roots filled, with few recurrences of abscesses or open fistulæ.

My method of procedure is as follows for Class One: In the management of this class of pulpless teeth there are three things that I invariably insist upon and carry out faithfully. First, complete disinfection; nothing is allowed to enter the pulp chamber or canals that is not placed there by my own hands, by means of clean and sterilized instruments; water and saliva are rigidly excluded. Second, no powerful coagulators of albumen are introduced into the pulp chamber or canals prior to the removal of the pulp. Third, the root filling material must be innocuous, tabooing the incorporation of antiseptics or disinfectants as a component of the filling material. If I have destroyed the pulp by any of the methods above spoken of, immediate extirpation under a local or other anæsthetic, by the cautery, wooden point (a barbarous method), or if it is in a recently fractured tooth, I assist the cessation of hemorrhage, then swab or syringe the pulp chamber and canals with equal parts of peroxide of hydrogen and a one-thousandth solution of bichloride of mercury until they are free from blood and serum. The root is then desiccated with absolute alcohol and heated air, and filled at once. If I have destroyed the pulp with arsenic or other corrosive, at the end of two days or therabouts the corrosive is removed under antiseptic precautions, keeping the tooth and cavity dry while a dressing of tannin and glycerine is applied to the devitalized pulp, first puncturing it to allow the congested blood to escape. This dressing is covered with gutta-percha, through which two or three perforations are made with a pointed instrument to allow any confined air to escape. This is allowed to remain eight days, when the dressing is removed. The rubber-dam is adjusted over the tooth, and the pulp is extirpated with suitable sterilized broaches. It will be found healthy and much reduced in bulk by this treatment. The pulp chamber and canal or canals are to be washed and swabbed with the peroxide and bichloride solution above mentioned, the root desiccated and filled at once. The roots are not reamed or drilled, but access is obtained by freely opening the pulp chamber.

Class Two. In the management of this class of pulpless teeth we generally find little trouble in diagnosis from the observance of three conditions, one of which is always present, *i. e.*, encystment of the apex; a dormant or cold abscess; or a fistulous tract leading from the end of the root externally or occasional lifting the peridental membrane from the root, and pus found escaping at gingival margin.

*Encystment.*—This may be suspected if the tooth has been free from tenderness in mastication, or when it is unaffected by thermal change, and the canals are dry when opened into for the first time. Absence of odor is also to be noted. Treatment: disinfectant dressing and immediate root filling.

*Cold or dormant Abscess.*—The rubber-dam being adjusted and the tooth and drill bathed in terebene or terpinol, the pulp chamber is entered. The contents of the chamber and canal are at once bathed with a penetrating disinfectant, which is allowed to remain for a few minutes. The debris is removed and the pus or watery exudation is drained through the canal. The peroxide and bichloride solution aforementioned is then injected or pumped into the territory beyond the apex of the root until it will come out clean; the roots are not drilled or reamed unless they are carious. A slender strand of cotton wool, saturated with terpinol or other essential oils, is packed in the root, not very lightly, and the pulp chamber filled with dry cotton, over which is packed some soft gutta-percha. This is perforated to allow air or any mephitic gases to escape. The patient is dismissed for ten days. On his reappearance the dressing is removed, the tooth being left dry. In most cases it will be found that the cold abscess has disappeared; this is determined by the absence of pus or moisture, and freedom from tenderness on percussion of the tooth. Should moisture exude into the canal the treatment must be repeated. The secret of the value of a root-dressing composed of one or more essential oils is this: At a temperature a little less than 95° F. camphors are slowly but certainly deposited from the oils, and vaporized almost immediately, so that the recently cleansed surface of a dormant abscess is stimulated to reproduce tissue which will replace that already dissipated by the distension of the peridental membrane and the formation of an abscess. These camphors are non-irritant disinfectants.

To introduce a powerful escharotic or coagulator of albumen in the treatment of a cold abscess not providing for drainage, would be most disastrous, and would generally lead to the formation of an acute abscess. It were far better practice to cut through the gum and alveolar process and provide an outlet at once than to attempt such a practice. The accidental forcing through the apex of a root, of any septic matter, is always to be avoided, and even the greatest care exercised by the immediate and indiscriminate root-fillers is not sufficient to prevent much suffering, and the occasional loss of a valuable tooth from inadequate disinfection. Time is always a factor in the destruction of microbes and their spores, and the disinfection of infective dentine is a matter of no small importance when it is hoped, by filling a root, that the operation will prove permanently successful. The future welfare of the cementum and pericementum is largely dependent on complete disinfection of the dentine prior to root-filling. The practice of leaving antiseptics and disinfectants within the root canal or pulp chamber as a safeguard against future trouble is delusive and is not founded on a knowledge of their potency in such situations

*Fistulæ.*—When a fistulous tract leads from the apex of a root to the gum or elsewhere, cleansing the canal of debris, disinfection of the dentine, and injection or pumping through the canal and fistula of eugenol, terebene, or five per cent, carbolic acid, and immediate root-filling, is generally permissable. When there are complications of caries or necrosis of the alveolar plate, surgical interference is required. In some cases of cold abscess, and more frequently when there is an open fistula, small blackish nodular deposits may be found around the apices of the affected roots, which it is needless to observe should be removed before the abscess will heal.

Class Three. Treatment: the same as for Class Two.

Class Four. In very many cases of pulpless teeth, from gradual exposure and death of the pulp, we find either encystment or cold abscess; more frequently the latter. This has already been sufficiently described in detail. When the pulp dies from mechanical abrasion or some form of erosion, we have been surprised to find that in nearly every instance the apices of the roots have been encysted, and such teeth in consequence are fit subjects for immediate root-filling when thoroughly disinfected.

Class Five. In the treatment of this class of pulpless teeth, when it is thought desirable to retain them in the mouth I have found that immediate root-filling is not to be practiced with impunity. The peridental membrane, remaining on the cementum, detached, or with only a loose hold upon it, is so feebly vital that the most careful stimulation and the retention of temporary dressing in the canals until the fistulæ have healed from the bottem outwards is most productive of good results. My usual practice is to remove debris and the pulp, cleansing the canals, as in Class One, then pack the roots tightly with threads of cotton wool, saturated with

Ol. Cinnamon - - - - m. xx,

Ol. Gaultheria, Terebene, - aa m. xxx, and seal the crown cavity with hard gutta-percha. This dressing is generally allowed to remain from one to six months when the root is filled permanently.

Class Six. In the management of this class of cases circumstances generally point to the necessity for temporary root dressings in order to prevent pain and swelling on the face. Whenever any uncertainty exists in the mind of the operator about the possible outcome of the hasty filling of a root, the interest of your client and second judgment will dictate the course to pursue, which may, and probably will be, the temporary dressing with a perforated gutta-percha covering. In the consideration of the various classes of pulpless teeth as they are met with in daily practice, I am not unmindful of possible complications; very many cases, indeed, are not seen by the surgeon until pain has driven the patient to him for aid. Certain relief is always afforded by drilling directly into the pulp chamber, supporting the tooth with the fingers, or producing torsion with a ligature held in the hand of an assistant. This operation is always to be preferred to drilling through the process, unless the patient has great fortitude, it is convenient to apply a local anæsthetic. If the swelling is great and the abscess can be reached conveniently with a bistoury, that operation will be indicated. After entering the pulp chamber, and gently removing the remains of the pulp, under antiseptic precautions, very great comfort may be assured the patient by painting the dried gum with equal parts of chloroform, tincture of aconite root, and tincture of iodine, and the immediate administration of 1-10 gr. pills of calcium sulphide every fifteen minutes until four or six have been taken, or thirty drop doses in water of fluid extract of Tonga every fifteen minutes. The drill hole should be stopped very loosely with iodoform cotton or cotton moistened with an essential oil, for two days, when all soreness will have disappeared and the root may be cleansed and dressed as mentioned in the consideration of Class Two, under the sub-healing of cold abscess. Occasionally pulpless teeth are elongated, and when the jaws are closed, pain is produced. This can be obviated by grinding the points or cusps of the molars and bicuspids, or other occluding surfaces.

I have no desire to lengthen this already long paper, but may I be permitted to observe that much of the success in the management of pulpless teeth is due to the thorough removal of all pulp tissue and other foreign matters, and the fidelity of filling the roots with a substance not porous or corruptible by the fluids of the mouth, or the products of microbes, and free from intermixed antiseptics? No dependence is to be placed in the permanency of incorporated antiseptics in root-fillings. I believe it to be a mistaken idea that only the apex is to be sealed, and would regard it as a step in advance, if all surgeons would abandon the stopping of roots with cotton, wool, silk or other vegetable fibre saturated with antiseptics, and adopt either an incorruptible oxychloride, oxyphosphate or gutta-percha as a root-filling. All care and skill exercised in the disinfection of dentine, and the treatment of abscesses goes for nought after the lapse of a few years, when reliance is placed on makeshifts of root-filling materials. Unless the root of a tooth is thoroughly well stopped by the filling, discoloration is not the least disfigurement of the anterior teeth, to say nothing of chronic peridental inflammation and the establishment of fistulæ and the recurrence of previously cured abscesses, with all the attendant evils and much suffering to the patient.—*British Journal.*

